# Long-term effects of local radiotherapy on growth and vertebral features in children with high-risk neuroblastoma

**DOI:** 10.1186/s12887-024-04813-z

**Published:** 2024-05-30

**Authors:** Kyungmi Yang, Joong Hyun Ahn, Sook-Young Woo, Sang Hoon Jung, Ki Woong Sung, Ji Won Lee, Do Hoon Lim

**Affiliations:** 1 Department of Radiation Oncology, Samsung Medical Center, Sungkyunkwan University School of Medicine, 81 Irwon-Ro, Gangnam-Gu, Seoul, Korea; 2 Biomedical Statistics Center, Data Science Research Institute, Research Institute for Future Medicine, Samsung Medical Center, Seoul, Korea; 3 Department of Pediatrics, Samsung Medical Center, Sungkyunkwan University School of Medicine, Seoul, Korea

**Keywords:** Growth restriction, Radiotherapy, Proton beam therapy, Neuroblastoma

## Abstract

**Background:**

To evaluate the effects of local radiotherapy (RT) on growth, we evaluated the chronological growth profiles and vertebral features of children with high-risk neuroblastoma.

**Methods:**

Thirty-eight children who received local photon or proton beam therapy to the abdomen or retroperitoneum between January 2014 and September 2019 were included. Simple radiography of the thoracolumbar spine was performed before and every year after RT. The height and vertical length of the irradiated vertebral bodies (VBs) compared with the unirradiated VBs (vertebral body ratio, VBR) were analyzed using the linear mixed model. Shape feature analysis was performed to compare the irradiated and unirradiated vertebrae.

**Results:**

The follow-up was a median of 53.5 months (range, 21–81 months) after RT. A decline in height z-scores was mainly found in the early phase after treatment. In the linear mixed model with height, the initial height (fixed, *p* < 0.001), sex (time interaction, *p* = 0.008), endocrine dysfunction (time interaction, 0.019), and age at diagnosis (fixed and time interaction, both *p* = 0*.*002) were significant. Unlike the trend in height, the change in VBR (ΔVBR) decreased gradually (*p* < 0.001). The ΔVBR in the group that received more than 30 Gy decreased more than in the group that received smaller doses. In the shape feature analysis, the irradiated VBs changed to a more irregular surface that were neither round nor rectangular.

**Conclusion:**

The irradiated VBs in children were gradually restricted compared to the unirradiated VBs in long-term follow-up, and higher RT doses were significantly affected. Radiation-induced irregular features of VBs were observed.

**Supplementary Information:**

The online version contains supplementary material available at 10.1186/s12887-024-04813-z.

## Background

Neuroblastoma is the most common extracranial solid tumor in children [1, 2]. The median age at diagnosis is approximately two years, with most cases diagnosed before five years old [3]. It is an embryonic cancer that arises from neural crest stem cells, and the common primary sites are the adrenal medulla and paraspinal ganglia in the abdomen and pelvis [4]. Its clinical course is heterogeneous, with treatment based on risk. Treatment for high-risk neuroblastoma has recently progressed with high-dose chemotherapy, autologous stem cell transplantation (HDCT/auto-SCT), and external beam radiotherapy (RT) [2, 5]. The clinical outcomes have improved gradually, and the five-year overall survival rate has been reported to be over 70% [6]. However, toxicity after treatment is unavoidable [6]. Some children have experienced growth impairment due to endocrine dysfunction or the effect of RT. In our protocol, total body irradiation (TBI) has been replaced by metaiodobenzylguanidine (MIBG) therapy, with RT used as localized therapy for primary sites and/or metastatic lesions. Growth impairment has improved following the protocol change [7]. However, a decreasing trend in the z-score of height has been reported.

We aimed to evaluate the chronological effect of local RT on growth and spinal features on X-rays in children with neuroblastoma.

## Methods

### Patients

We retrospectively reviewed the medical records of children with high-risk neuroblastoma who were treated with local RT between January 2014 and September 2019. The high-risk group was defined as having at least one of the following risk factors: International Neuroblastoma Staging System (INSS) stage 4 with an age at diagnosis of one year or older and N-myc gene amplification regardless of stage [8]. Children were irradiated in the abdomen or retroperitoneum as a primary site, and simple radiography of the thoracolumbar (T-L) spine was taken before and every year after RT to check the growth and alignment of the spine. To focus on the impact on spinal deformities or growth related to the T-L spine, the exclusion criteria were: (1) boys over 12 years and girls over ten years, (2) the mediastinal origin of neuroblastoma, or (3) patients irradiated out of the T-L spine. Ultimately, 38 children were included.

### Treatment of neuroblastoma

The treatment for high-risk neuroblastoma was based on our protocol [8, 9]. Briefly, treatment consisted of nine cycles of induction chemotherapy with surgery after six cycles of chemotherapy, tandem HDCT/auto-SCT, local RT, differentiation therapy with 13-cis-retinoic acid, and immunotherapy with interleukin-2. Most patients in this study were treated using the up-to-date protocol SMC NB-2014 (NCT02771743), which modified the doses of the first HDCT and HD-MIBG according to the tumor status before each HDCT/auto-SCT based on ^123^I-MIBG or ^18^FDG-PET uptake. In the NB-2009 protocol, TBI was not routinely performed because of toxicity and growth impairment [6].

Local RT was administered mainly to the primary tumor site approximately six weeks after the second HDCT/auto-SCT. From January 2016, our institute started proton beam therapy (PBT) using a posterior beam, which results in less exposure to the anterior part of the body. We previously used three-dimensional conformal radiotherapy (3D-CRT) with anterior–posterior parallel opposite fields. The RT dose was 15.0 Gy in 1.5 Gy per fraction for patients without residual tumors and 21.6 ~ 36.0 Gy in 1.8 Gy per fraction for patients with residual tumors before RT. The target volume was defined as the preoperative/postchemotherapy primary tumor bed (clinical target volume), including residual tumor (gross tumor volume), if present, with a 1.0 to 1.5 cm margin for the planning target volume. If a vertebral body (VB) was partially included in the target volume, the body was encompassed entirely up to at least 15 Gy because of concerns about inhomogeneous growth, regardless of photon or PBT. We evaluated the VB dose based on the prescription dose. Most irradiated VBs (iVBs) were exposed with doses exceeding 15 Gy, and in cases where the prescription dose exceeded 15 Gy, the anterior aspect of the VB had radiation exposure consistent with the prescribed dose.

### Analysis of growth and vertebrae

The growth profiles of the children were regularly recorded at the time of diagnosis and at every visit. We analyzed height, weight, and body mass index (BMI) at the time of diagnosis (t_i_), immediately prior to RT (pre-RT or t_r_), and at annual follow-ups with T-L spine X-ray (t_1_-t_5_). The parameters are expressed as z-scores or percentiles of the same age based on the 2017 Korean National Growth Charts [10].

To evaluate the effect of RT on the spine, the mid-vertical lengths of iVBs and unirradiated VBs (uVBs) were measured on the lateral view of the T-L spine X-ray. The length of iVB was measured for all the VBs in the RT target volume. The length of uVBs was measured at the same number of vertebrae above the RT field, excluding at least one vertebra directly above the RT field. The vertebral body ratio (VBR) at a certain time was defined as the length per iVBs divided by the length per uVB. The change in VBR (ΔVBR, %) at time t_i_ was defined as [(VBR at t_i_ -VBR at t_0_) / VBR at t_0_] × 100.

### Shape feature analysis

To compare the sectional shapes of iVB and uVB, two-dimensional shape feature extraction was performed on anteroposterior (AP) and lateral views of simple X-rays to the T-L spine (Supplementary Fig. S1). For 14 patients who were followed up for five years after RT, one radiation oncologist contoured the VBs of L1 and T7, which were representatives of iVB and uVB, respectively, on the X-ray films taken before RT (pre-RT) and at the five-year follow-up (post-RT): L1, representing an area included RT field and T7, representing an out-field area in all patients. MIM (version 6.4.9, MIM software, OH, US) was used for contouring. The films and contours were transferred to an in-house program as DICOM files. The following feature parameters were extracted: (1) area, perimeter, perimeter to the area, and sphericity from a contour; (2) convex area, convex perimeter, convexity, solidity, and elongation from a convex hull, which defines the smallest convex region, including a contour; and (3) eccentricity and rectangularity from a minimum bounding rectangle [11, 12].

### Statistical analysis

Linear mixed model analysis was performed on repeatedly measured data, such as height, weight, BMI, and VBR. The pre-RT and five-year follow-up shape features were compared using the paired t-test. Multiple comparison was adjusted by the Bonferroni method. Statistical analyses were performed using SPSS version 27 (SPSS Inc., IBM Company, Chicago, IL, USA). Statistical significance was set at *p* < 0.05. Bonferroni correction was applied for multiple comparisons.

## Results

### Patient characteristics

The patient characteristics are summarized in the Supplementary Table S1. The median age at diagnosis was 36.5 months (range: 0–128 months). The study included 23 males (60.5%). Regarding the histologic type, most patients had neuroblastoma (84.2%), except six with ganglioneuroblastoma. Twenty-five (65.8%) patients underwent surgery on the primary tumors, and most patients were treated with MIBG (84.2%).

Local RT was performed at a median age of 50.5 months (13–142 months). The median interval from diagnosis to RT was 14 months (7–17 months). There was a median number of five iVBs (3–9), and the RT doses were 15, 25.2, 30.6, and 36.0 Gy in 17 (44.7%), five (13.2%), five (13.2%), and 11 (28.9%), respectively. PBT was performed in 25 patients (65.8%).

### Events and complications related to growth

The median follow-ups were 66.5 (range, 38–96) and 53.5 (range, 21–81) months from diagnosis and RT, respectively. One child (2.6%) died from osteosarcoma, and six patients (15.8%) experienced disease progression (four distant metastases and two in-field recurrences with distant metastasis). Endocrine dysfunction, hypothyroidism, growth hormone deficiency (GHD), and sex hormone deficiency were observed in 16 (42.1%), 14 (36.8%), three (7.9%), and one patient (2.6%), respectively. Two children were diagnosed with hypothyroidism and GHD. All patients were treated with appropriate therapies, including hormone supplementation.

**Fig. 1:**
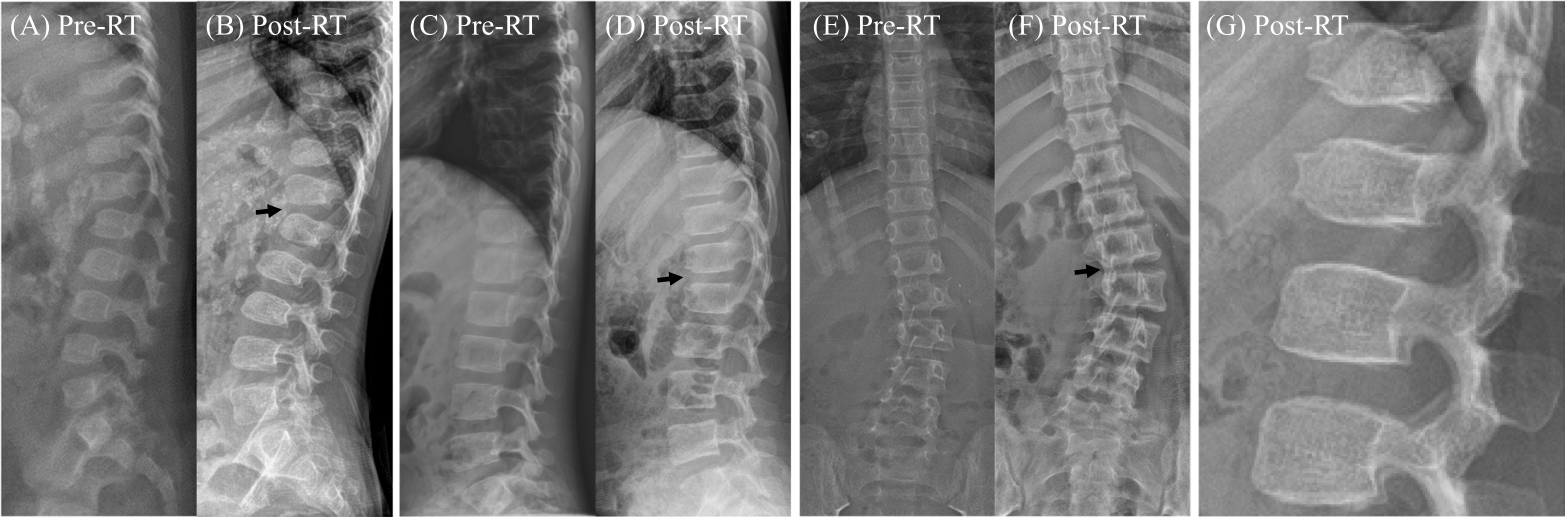
Pre-RT and post-RT (five years after RT) thoracolumbar X-ray films in four cases. The first child diagnosed with neuroblastoma at five months showed kyphotic features before RT (**A**), which was similarly observed post-RT X-ray (**B**). Mild kyphosis was newly observed on post-RT film (**C** and **D**) for the second child treated with RT at 87 months. The third case with RT at 106 months already showed scoliosis before RT (**E**), and it progressed at five years after RT (**F**). Irradiated upper vertebrae (T12-L1) were more irregular than unirradiated lower vertebrae (L2-L3) five years after RT in the youngest children who were diagnosed with neuroblastoma at birth and treated with RT at 13 months (**G**)

Abnormalities in spine arrangement were observed in three patients (two kyphotic and one scoliotic; Fig. 1). The features in two patients were already shown before RT (Fig. 1A, B, E and F). The patient with scoliosis (Fig. 1E and F) seemed to be progressive. None of the patients had any symptoms that required orthopedic treatment.

### Growth profiles

**Fig. 2:**
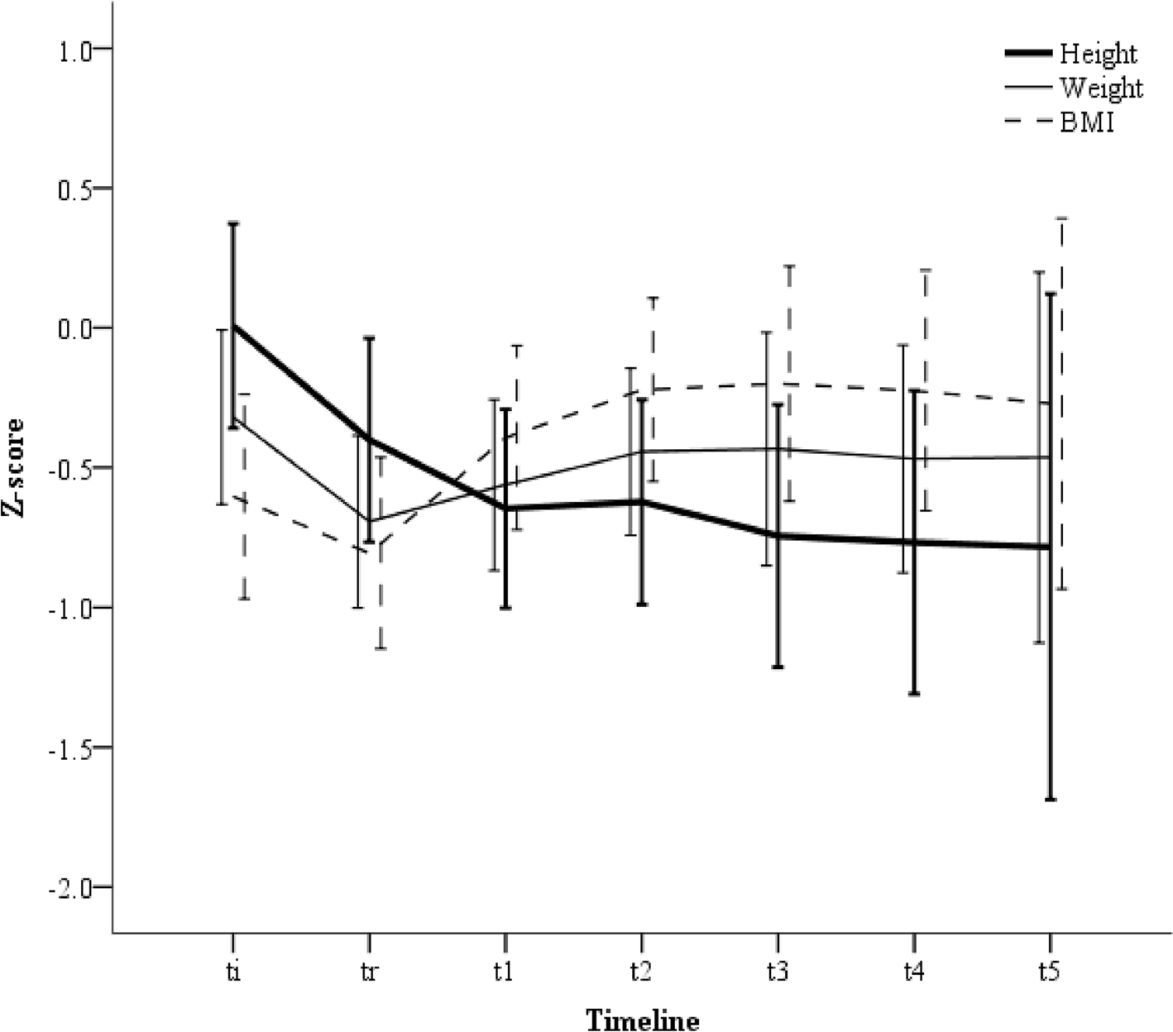
Chronological changes of the height, weight, and BMI z-scores from the time of diagnosis; the lines and the bars, the mean of z-scores, and the 95% confidential interval, respectively; *t*_*i*_ time point of initial diagnosis, *t*_*r*_ time point immediately preceding the start of radiation therapy, *t*_*1-4*_ time points of the one- to four-year interval follow-ups after radiotherapy

Repeated measured z-scores for height, weight, and BMI are shown in Fig. 2, and the linear mixed model analysis results are shown in Table 1. Overall, the trends in the three parameters were significant (*p* < 0.001, *p* = 0.001, and *p* < 0.001, respectively). The height was initially 0.007 and decreased gradually. However, the decrease from t_1_ to t_5_ was not statistically significant. The z-score of the weight started at -0.320, decreased at t_r_ (*p* < 0.001), and then recovered at t_1_.

**Table 1:** Z-scores of the height, weight, and BMI from diagnosis (timeline t_i_), and a comparison between two time points of each year. (A) The analysis used the linear mixed model, and the *p*-values for subgroup comparison were adjusted using Bonferroni correction. (B) Factors related to the z-score of height by the linear mixed model

**(A)**	**Timeline**	**t**_**i**_	**t**_**r**_	**t**_**1**_	**t**_**2**_	**t**_**3**_	**t**_**4**_	**t**_**5**_	***p* for time**
Height (est. z-score)		0.007	-0.401	-0.647	-0.624	-0.788	-0.793	-0.777	< 0.001
Comparison (*p*-value)	t_i_	-	< 0.001	< 0.001	< 0.001	< 0.001	< 0.001	0.001	
	t_r_	-	-	0.005	0.341	0.029	0.166	0.724	
	t_1_	-	-	-	1.000	1.000	1.000	1.000	
	t_2_	-	-	-	-	0.818	1.000	1.000	
	t_3_	-	-	-	-	-	1.000	1.000	
	t_4_	-	-	-	-	-	-	1.000	
Weight (est. z-score)		-0.320	-0.694	-0.562	-0.443	-0.393	-0.376	-0.384	0.001
Comparison (*p*-value)	t_i_	-	< 0.001	0.583	1.000	1.000	1.000	1.000	
	t_r_	-	-	1.000	0.480	0.684	1.000	1.000	
	t_1_	-	-	-	1.000	1.000	1.000	1.000	
	t_2_	-	-	-	-	1.000	1.000	1.000	
	t_3_	-	-	-	-	-	1.000	1.000	
	t_4_						-	1.000	
BMI (est. z-score)		-0.604	-0.806	-0.394	-0.222	-0.108	-0.103	-0.161	< 0.001
Comparison (*p*-value)	t_i_	-	1.000	1.000	0.197	0.075	0.186	0.872	
	t_r_	-	-	0.002	< 0.001	0.001	0.005	0.068	
	t_1_	-	-	-	1.000	1.000	1.000	1.000	
	t_2_	-	-	-	-	1.000	1.000	1.000	
	t_3_	-	-	-	-	-	1.000	1.000	
	t_4_	-	-	-	-	-	-	1.000	
**(B) Variables**			**Univariable (*p*-values)**				**Multivariable (*p*-values)**		
			**Time**	**Variable**	**Interaction**		**Time**	**Variable**	**Interaction**
Height at diagnosis (%)	< 30 vs. ≥ 30		< 0.001	< 0.001	0.155		< 0.001	< 0.001	0.119
Weight at diagnosis (%)	< 5 vs. ≥ 5		< 0.001	0.019	0.056		< 0.001	0.059	0.607
Age at diagnosis (months)	< 36 vs. ≥ 36		< 0.001	0.163	< 0.001		< 0.001	0.002	0.002
Sex	F vs. M		< 0.001	0.103	0.005		< 0.001	0.158	0.008
Surgery	Yes vs. No		< 0.001	0.158	0.907				
MIBG therapy	Yes vs. No		< 0.001	0.269	0.666				
No. of irradiated VB	3–4 vs. ≥ 5		< 0.001	0.567	0.024		< 0.001	0.943	0.274
RT dose, Gy	15.0–25.2 vs. 30.6–36.0		< 0.001	0.432	0.899				
RT type	Photon vs. Proton		< 0.001	0.627	0.853				
Endocrine dysfunction	Yes vs. No		< 0.001	0.422	0.004		< 0.001	0.187	0.019
Progression	Yes vs. No		0.001	0.857	0.304				

*BMI* body mass index, *MIBG* metaiodobenzylguanidine, *VB* vertebral body, *RT* radiotherapy, *t*_*i*_ time point of initial diagnosis, *t*_*r*_ time point immediately preceding the start of radiation therapy, *t*_*1-4*_ time points of the one- to four-year interval follow-ups after radiotherapy

Linear mixed analysis was performed to identify the significant factors related to height (Table 1). From the univariate analysis, the initial height and weight were significant when the time factor was fixed (*p* < 0.001 and *p* = 0.019, respectively). Regarding the time-variable interactions, age, sex, number of iVBs, and endocrine dysfunction were significant (*p* < 0.001, *p* = 0.005, 0.024, and 0.004, respectively). In the multivariable analysis of the factors with *p* < 0.1 from the univariable analysis, the initial height was associated with the z-score fixed by time (*p* < 0.001), and sex and endocrine dysfunction were significant in the time-variable interactions (*p* = 0*.*008, and 0.019, respectively). Age at diagnosis was related to the z-score trend in the time-fixed level and time-variable interactions (all *p* = 0*.*002).

### Trends in the vertebral body ratio

**Fig. 3:**
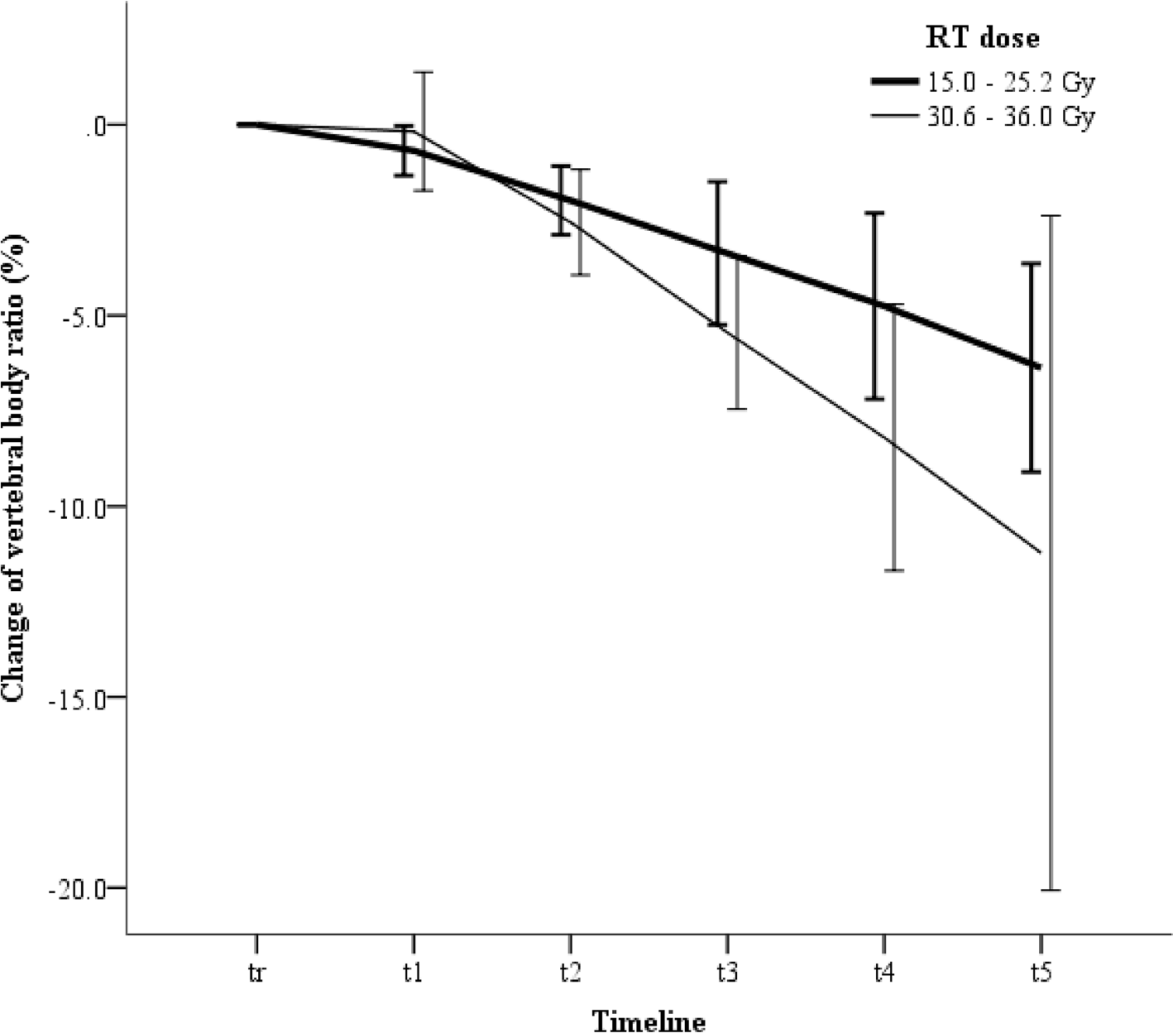
Changes in the vertebral body ratio associated with radiation dose; *t*_*i*_ time point of initial diagnosis, *t*_*r*_ time point immediately preceding the start of radiation therapy, *t*_*1-4*_ time points of the one- to four-year interval follow-ups after radiotherapy

Similar to the growth profiles, the VBR was also analyzed using a linear mixed model (Table 2). The VBR showed a significant declining trend (*p* < 0.001). The VBR started to decrease significantly from t_2_ compared to the previous t_r_ or t_1_ (all *p*-values < 0.005 from t_2_) (Table 2). In the multivariable analysis based on the variables with *p* < 0.001 in the univariate analysis, only the RT dose was significant in both time-fixed level and time-variable interactions (*p* = 0*.*007 and 0.013). The ΔVBR in the group with 30.6 Gy or 36 Gy decreased more than that in the group with 15 Gy or 25.2 Gy (Fig. 3).

**Table 2:** Changes of the vertebral body ratio (Δ VBR, %) from pre-RT (timeline t_r_) and the comparison between two time points of each year. (A) The analysis used the linear mixed model, and the *p*-values for subgroup comparison were adjusted using Bonferroni correction. (B) Factors related to ΔVBR by the linear mixed model

**(A)**	**Timeline**	**t**_**r**_	**t**_**1**_	**t**_**2**_	**t**_**3**_	**t**_**4**_	**t**_**5**_	***p* for time**
ΔVBR (%)		0	-0.467	-2.203	-4.132	-5.944	-7.793	< 0.001
Comparison (*p*-value)	T_r_	-	1.000	< 0.001	< 0.001	< 0.001	< 0.001	
	T_1_	-	-	1.000	< 0.001	< 0.001	< 0.001	
	T_2_	-	-	-	< 0.001	< 0.001	< 0.001	
	T_3_	-	-	-	-	0.008	< 0.001	
	T_4_	-	-	-	-	-	0.011	
**(B)** **Variables**			**Univariable (*p*-values)**			**Multivariable (*p*-values)**		
			**Time**	**Variable**	**Interaction**	**Time**	**Variable**	**Interaction**
Height at diagnosis (%)	< 30 vs. ≥ 30		< 0.001	0.584	0.947			
Weight at diagnosis (%)	< 5 vs. ≥ 5		< 0.001	0.599	0.100	< 0.001	0.971	0.307
Age at diagnosis	< 36 vs. ≥ 36		< 0.001	0.548	0.427			
Sex	F vs. M		< 0.001	0.164	0.247			
Surgery	Yes vs. No		< 0.001	0.450	0.185			
MIBG therapy	Yes vs. No		< 0.001	0.804	0.018	< 0.001	0.906	0.066
No. of VB	3–4 vs. ≥ 5		< 0.001	0.296	0.362			
RT dose, Gy	15.0–25.2 vs. 30.6–36.0		< 0.001	0.006	0.006	< 0.001	0.007	0.013
RT type	Photon vs. Proton		< 0.001	0.583	0.371			
Endocrine dysfunction	Yes vs. No		< 0.001	0.104	0.114			
Progression	Yes vs. No		< 0.001	0.677	0.984			

*VB* vertebral body, *VBR* vertebral body ratio, *MIBG* metaiodobenzylguanidine, *RT* radiotherapy

### Change of shape feature

Fourteen patients underwent post-RT X-ray films at five years, and the changes in the shape feature parameters were calculated from the pre-RT X-ray (Table 3). The increase in area in L1 was less than that in T7 in both views (*p* = 0*.*002 and *p* = 0.011, respectively). The perimeter in L1 of the lateral view was not different from that in T7 (*p* = 0*.*399), which led to a smaller decrease in the perimeter of the area in L1 (*p* < 0.001). Sphericity decreased in L1 but increased in T7 (*p* = 0*.*023 and *p* < 0.001). The results imply that the body of L1 grew less but had a more irregular surface than T7. Regarding the parameters of the convex hull, the convex area, convex perimeter, and elongation in L1 increased less than those in T7. Convexity and solidity were not different in L1 and T1 in the AP view but were in the lateral view. Convexity and solidity in L1 in the lateral view decreased (*p* = 0*.*001 and 0.017, respectively), indicating that the surface of the contour was more irregular and less round. Regarding the minimum bounding rectangle parameters, most comparisons were significant, except for rectangularity in the AP view. After RT, the shape of L1 had a larger difference in the long and short axes than that of T7 and had changed away from the rectangular shape in the lateral view.

**Table 3:** Comparison of the five-year change after radiotherapy between irradiated and unirradiated vertebral bodies (L1 and T7) from the shape feature analysis

Shape	Parameter, Δ (%)	AP			LAT		
		**L1**	**T7**	***p*-value**	**L1**	**T7**	***p*-value**
(1) Contour	Area	88.4 ± 31.4	113.2 ± 37.7	0.002	102.7 ± 40.9	125.5 ± 48.3	0.011
	Perimeter	37.4 ± 10.7	43.5 ± 11.7	0.021	46.5 ± 15.7	48.9 ± 14.8	0.399
	Perimeter to area	-26.1 ± 6.4	-31.8 ± 5.8	0.001	-26.5 ± 7.2	-32.8 ± 6.3	< 0.001
	Sphericity^a^	-0.4 ± 2.3	1.5 ± 2.5	0.023	-3.2 ± 2.5	0.4 ± 2.6	< 0.001
(2) Convex hull	Convex area	90.0 ± 32.3	114.8 ± 36.8	0.023	104.8 ± 42.0	125.1 ± 47.2	0.047
	Convex perimeter	36.5 ± 11.0	41.8 ± 11.0	0.001	44.2 ± 15.5	49.5 ± 14.6	0.022
	Convexity^b^	-0.6 ± 1.2	-1.1 ± 1.9	0.457	-1.6 ± 1.5	0.4 ± 1.4	0.001
	Solidity^c^	-0.8 ± 1.4	-0.8 ± 1.4	0.944	-1.0 ± 1.5	0.1 ± 1.0	0.017
	Elongation^d^	3.1 ± 6.6	9.9 ± 9.3	0.022	-4.0 ± 5.6	4.2 ± 10.1	0.013
(3) Minimum bounding rectangle	Eccentricity^e^	2.5 ± 3.9	11.2 ± 5.2	< 0.001	-3.7 ± 3.9	2.9 ± 6.6	0.009
	Rectangularity^f^	-0.9 ± 4.3	-1.0 ± 3.6	0.930	-1.4 ± 3.2	2.4 ± 2.7	0.001

*AP* anteroposterior view, *LAT* lateral view

^a^Sphericity = perimeter of a round having the same area as the contour/perimeter of the contour

^b^Convexity = perimeter of the convex hull/perimeter of contour

^c^Solidity = area of the contour/area of the convex hull

^d^Elongation = the length of the major axis of the convex hull/the length of the minor axis of the convex hull

^e^Eccentricity = the length of the minor axis of the minimum bounding rectangle/the length of the major axis of the minimum bounding rectangle; and

^f^Rectangularity = the area of the contour/the area of the minimum bounding rectangle

## Discussion

We chronologically analyzed the growth of prepubertal children after treatment with neuroblastoma, including local RT. Similar to previous studies [7, 12], the z-scores of height decreased sharply from the diagnosis, and the slope became smooth from the second year. The z-score of weight decreased less than that of height and recovered slowly after treatment. This means that while the disease and treatment affect the growth restriction temporarily, the loss of height cannot be recovered despite appropriate nutritional status. The growth of pediatric patients with cancer has been reported to be affected by hormonal and non-hormonal factors [13]. Endocrine problems frequently occur in 40–60% of pediatric patients with cancer after treatment, and the main endocrine disturbances are disorders of the hypothalamic-pituitary axis, including GHD and thyroid dysfunction [14]. Chemotherapy and cranial irradiation had been the main treatment options [15]. These hormonal problems were found to be significant for growth impairment despite the absence of TBI or cranial RT and adequate supplements. Non-hormonal factors include age at the time of treatment, parental height, physical activity, nutrition, and damage to the growth plate from surgery or RT [13]. Similar to the results of this study, initial growth status and age at diagnosis were also significant factors in the z-score height.

In association with RT, the irradiated volume, total dose, or fraction size can affect growth [16]. The number of VBs and TBI were associated with a lower height percentile in our previous study [12]. However, the number of VBs was significant only in the univariate analysis. The estimated VBR change over five years was -7.793%. For the five-year follow-up after RT in 14 children, the vertical length per iVB and uVB grew from 21.4 mm to 25.3 mm and 16.5 mm to 21.4 mm, respectively. The restricted length of iVB was estimated to be approximately 2.5 mm per VB. It is thought that a loss of growth in a median of five vertebrae would have a limited effect on overall height.

The higher the RT dose, the greater its effect on spinal growth [16–19]. The effect of the RT dose varied according to age at RT and can be critical for growth in children aged zero to two years, even at less than 10 Gy. In children aged two to six years, it showed a substantial effect at > 15 Gy. For prepubertal patients aged six years or older, the effect of RT might cause damage at 35 Gy or higher, and the negative effect may be less but possible at between 15 and 35 Gy. Although the approximate limit of the RT dose is known, the cut-off value may be unclear. Therefore, the European Society for Pediatric Oncology radiotherapy working group recommended a vertebral dose gradient within 5 Gy for prepubertal children aged two years or older, avoiding a dose of more than 20 Gy to seven or more thoracic vertebrae if possible [16]. At our institute, RT targets for paravertebral sites in prepubertal patients include all VBs with at least 15 Gy. We observed that the growth restriction of iVB at more than 30 Gy was more evident than at relatively low doses. Therefore, although the intensity of vertebral damage might differ between patients, bony growth restriction in the RT field seems impossible to avoid after local RT with ≥ 15 Gy. Therefore, we should be careful in deciding the RT dose and irradiated volume.

PBT as an RT modality was not significantly associated with height restriction. PBT has been preferred for pediatric patients with cancer because it has shown potential benefits, such as lower secondary malignancy [20] and better hematologic outcomes after RT [21]. PBT can spare the anterior part of the body when locally irradiated using posterior beams. However, little is known about the effect of PBT compared to X-ray therapy in relation to height restriction. A study that followed patients for a median of 13.9 months after PBT reported a decrease in the growth rate of approximately 23% at 15 Gy and 60% at 30 Gy [22]. However, the effect of PBT was difficult to ascertain in this study because only growth at one point over one to two years was observed, and no patients underwent X-ray therapy. In our patients who underwent PBT or photon therapy and were followed up for a longer time, PBT was found to have a similar effect on height or vertebral growth as X-ray therapy. Nevertheless, a longer follow-up period is required.

A previous study by our institute reported on the shape change of iVBs in the axial view on magnetic resonance imaging [12]. The signal change of the irradiated vertebrae in the T1 weighted image and the roundness in the T2 weighted image decreased at a median of 86 months after RT. In this study, using simple radiography, we analyzed changes in the vertical length and contours of the AP and lateral views, which are thought to be more related to actual growth than the axial view. In addition to the decrease in the vertical length, the surface irregularity of iVB increased compared to uVB. iVB showed atypical features (Fig. 1G). In the lateral view of the radiograph, severe irregularities at the anterior borders and convexity were often observed. Growth plates, as secondary ossification centers, are located at the upper and lower ends of the VB, which is near the cartilage of discs [23]. After the growth plate of the VB was damaged from treatment, the surface appeared to have permanent scars as ossification, although the volume of iVB increased gradually. Finally, iVB exhibited an irregular and bulging shape at the top and bottom. It can be suggested that the primary ossification center in the middle of the VB is less sensitive to RT than the superior and inferior growth plates as secondary ossification centers [16]. Researchers have suggested a direct effect on chondroblasts [24] and microvascular damage [25]. Blood supply might be better in the middle of the bone than at the edges, which might be an advantage for the primary ossification center. Because of the primary center, the radiation dose effect may be complicated but sometimes obvious at higher doses [26].

This study had several limitations. Because this was a retrospective, single-center study with a small sample, detailed statistical verification of the factors was challenging. A simple X-ray film was not obtained every year for some patients, and several missing values were adjusted using a linear mixed model; After RT, X-ray films were taken in all patients at least for 2 years, but data were only obtained in 71.1% at 3 years, 55.3% at 4 years, and 36.8% at 5 years. As one of the ambiguities in this study, the VB dose was not clinically constrained and there was also dose gradients within the VB. Therefore, the VB dose was analyzed by substituting it with the prescription dose. Moreover, due to the relatively recent introduction of PBT, it was difficult to statistically confirm differences in shape among RT modalities based on feature analysis, with only 2 out of 14 patients having received PBT. Although vertical length included loss of length from the VB and abnormal arrangement of the spine [16], spinal deformities were not analyzed due to their rarity. If more comprehensive data are obtained, factors related to RT could be verified for growth restriction and RT-related orthopedic diseases.

## Conclusion

In children with neuroblastoma, local RT minimally affected height growth. However, iVB gradually grew significantly less than uVB, particularly at higher RT doses. We also observed highly irregular features of the vertebrae on the follow-up X-rays after RT.

### Supplementary Information

Supplementary Material 1.

## Data Availability

The datasets generated and/or analyzed during the current study are not publicly available due to concerns related to patient confidentiality and privacy. However, they are available from the corresponding author upon reasonable request.

## Abbreviations

AP: Anteroposterior
BMI: Body mass index
GH: Growth hormone
GHD: Growth hormone deficiency
IMPT: Intensity-modulated proton therapy
IMRT: Intensity-modulated radiotherapy
INSS: International Neuroblastoma Staging System
LAT: Lateral view
PBT: Proton beam therapy
PT: Proton therapy
RT: Radiotherapy
SCT: Stem cell transplantation
TBI: Total body irradiation
VB: Vertebral body
VBR: Vertebral body ratio
