# Supplementary material for: Long-term effects of local radiotherapy on growth and vertebral features in children with high-risk neuroblastoma
Source: BMC Pediatr. 2024 May 30;24:372. doi: 10.1186/s12887-024-04813-z (PMC11137931; doi:10.1186/s12887-024-04813-z)
Supplement: Supplementary file 1 — Supplementary Material 1. [file 12887_2024_4813_MOESM1_ESM.docx]

**Table S1**  Patient characteristics (*N* = 38)

| **Variables** |  | **%** |
| --- | --- | --- |
| Age at diagnosis, months | Median (range) | 36.5 (0-128) |
|  | 0-35 | 18 (47.4%) |
|  | ≥36 | 20 (52.6%) |
| Sex | Female | 15 (39.5%) |
|  | Male | 23 (60.5%) |
| Histology | Neuroblastoma | 32 (84.2%) |
|  | Ganglioneuroblastoma | 6 (15.8%) |
| INSS | 3 | 2 (5.3%) |
|  | 4 | 35 (92.1%) |
|  | 4S | 1 (2.6%) |
| N-myc amplification | Yes | 6 (15.8%) |
|  | No | 31 (81.6%) |
|  | Unknown | 1 (2.6%) |
| Surgery | Yes | 25 (65.8%) |
|  | No | 13 (34.2%) |
| MIBG therapy | Yes | 32 (84.2%) |
|  | No | 6 (15.8%) |
| No. of irradiated VB | Median (range) | 5 (3-9) |
|  | 3-4 | 15 (39.5%) |
|  | 5-6 | 20 (52.6%) |
|  | 8-9 | 3 (7.9%) |
| RT dose, Gy | Median (range) | 25.2 (15.0-36.0) |
|  | 15.0 | 17 (44.7%) |
|  | 25.2 | 5 (13.2%) |
|  | 30.6 | 5 (13.2%) |
|  | 36.0 | 11 (28.9%) |
| RT modality | 2D | 2 (5.3%) |
|  | 3D | 10 (26.3%) |
|  | IMRT | 1 (2.6%) |
|  | PT | 9 (23.7%) |
|  | IMPT | 16 (42.1%) |
| Endocrine dysfunction | Yes | 16 (42.1%) |
| - GH deficiency | Yes | 3 (7.9%) |
| - Hypothyroidism | Yes | 14 (36.8%) |

*INSS* International Neuroblastoma Staging System, *MIBG* metaiodobenzylguanidine therapy, *SCT* stem cell transplantation, *VB* vertebral body, *RT* radiotherapy, *2D* two-dimensional radiotherapy, *3D* three-dimensional conventional radiotherapy, *IMRT* intensity-modulated radiotherapy, *PT* three-dimensional proton therapy, *IMPT* intensity-modulated proton therapy, *GH* growth hormone

**
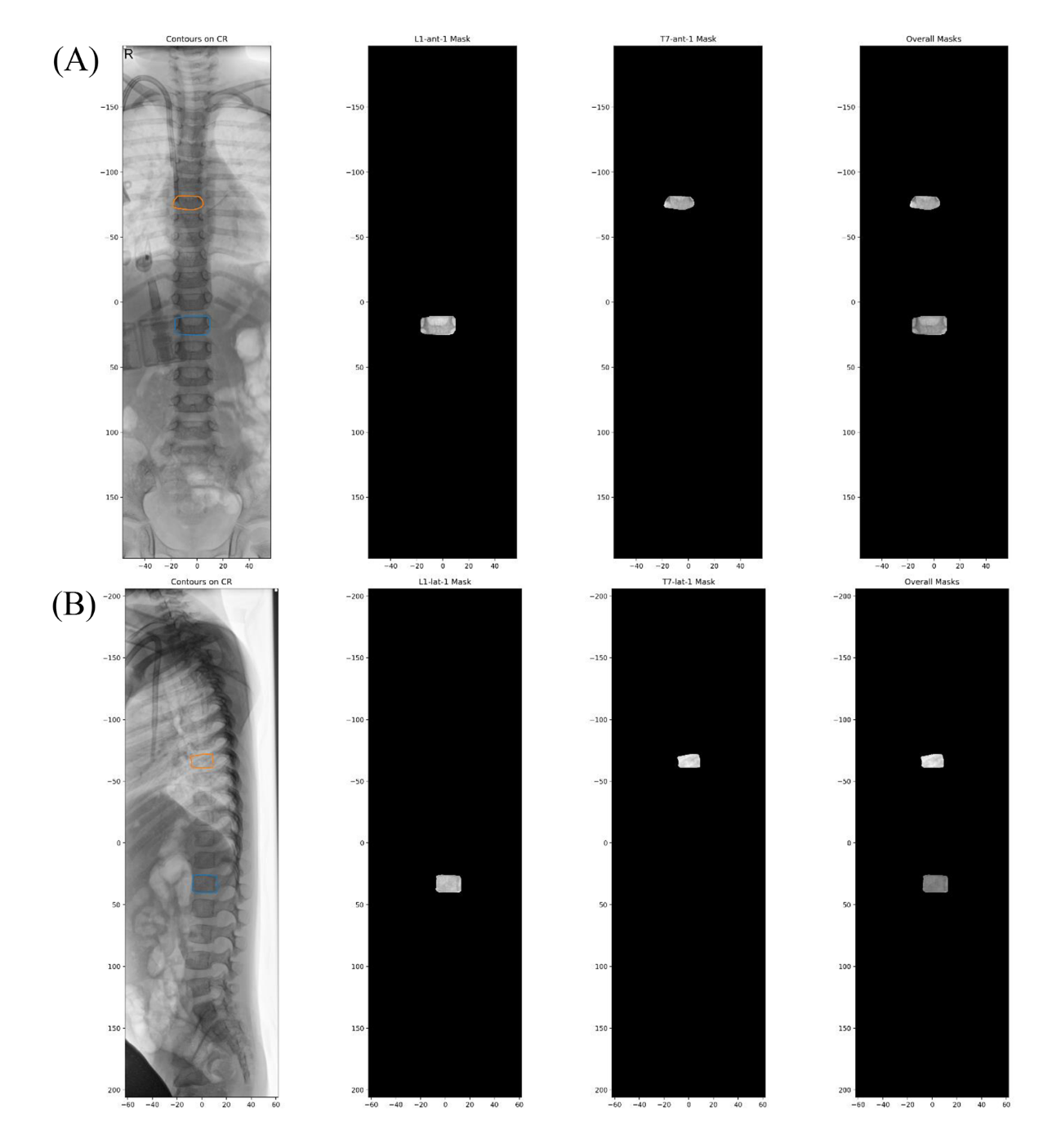
**

**Fig. S1** A sample of the feature extraction. Contours of T7 (orange color) and L1 (blue color) vertebral bodies on the anteroposterior (**A**) and lateral (**B**) views of thoracolumbar X-ray films.
